# Isolated Cilioretinal Artery Occlusion as an Initial Manifestation of Polycythemia Vera

**DOI:** 10.4103/0974-9233.65493

**Published:** 2010

**Authors:** Fouad Elasri, H. Souhail, K. Reda, S. Iferkhass, A. Idrissi, A. Naoumi, A. Oubaaz

**Affiliations:** Department of Ophthalmology, Mohammed V Military Teaching Hospital, University of King Mohammed V-Souissi, Rabat, Morocco

**Keywords:** Cilioretinal Artery, Occlusion, Polycythemia Vera

## Abstract

Isolated cilioretinal artery occlusion is a rarely reported initial manifestation of polycythemia vera. In this study, we reported a case of a 65-year-old man with polycythemia vera with cilioretinal artery occlusion as an initial manifestation.

## INTRODUCTION

A cilioretinal artery is present in up to 32% of eyes.^1^ Isolated cilioretinal artery occlusion is rare and can lead to monocular visual loss which is generally transient.^1^–^3^ However, isolated cilioretinal artery occlusion as an initial manifestation of polycythemia vera is extremely rare and is described in this study.

## CASE REPORT

**Figure 1:**
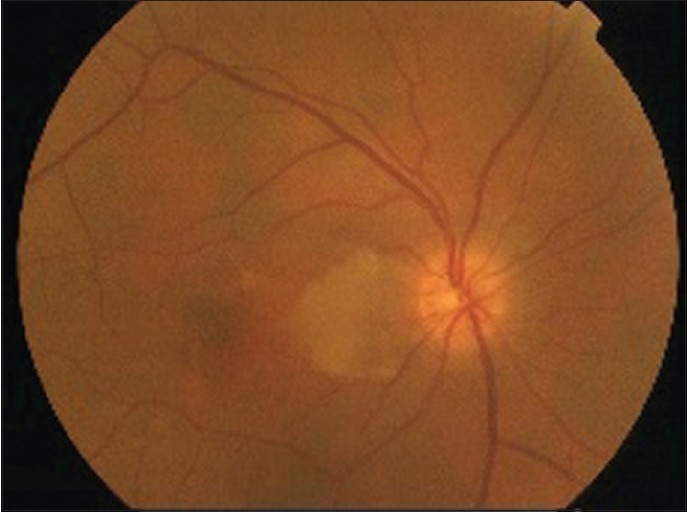
Fundus photographs showed an area of interpapillomacular retinal ischemia with whitish edges

**Figure 2:**
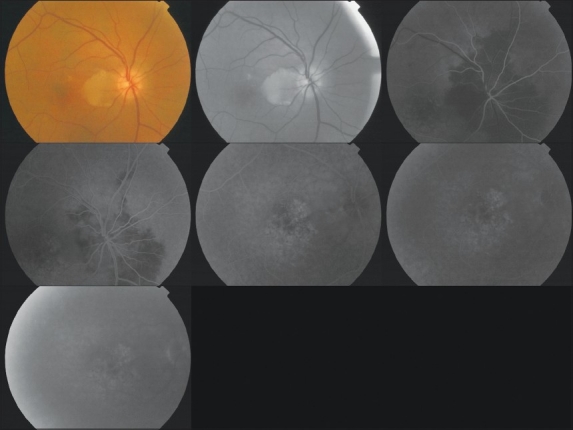
Fluorescein angiography showed masking of choroidal fluorescence due to retinal edema and delayed filling of the cilioretinal artery

**Figure 3:**
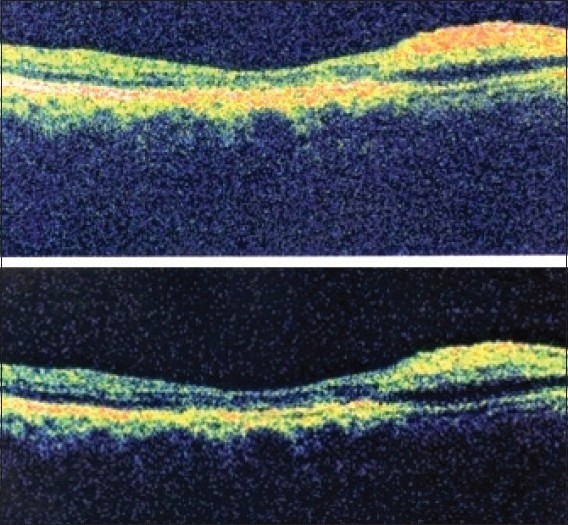
Optical coherence tomography showing the presence of a serous retinal detachment impinging the macula

A 65-year-old man presented with visual loss in the right eye with onset two days prior to presentation. On ophthalmic examination, visual acuity was 1/10 in the right eye and 10/10 in the left eye. The anterior segment examination was normal, and intraocular pressure was 14 mmHg in both eyes. Dilated fundus examination showed an area of interpapillomacular retinal ischemia with whitish edges [Figure 1]. The left eye was normal. Goldmann kinetic perimetry was not feasible due to the reduced vision in the right eye. Fluorescein angiography of the right eye showed masking of choroidal fluorescence due to retinal edema and delayed filling of the cilioretinal artery. The delayed circulation in the cilioretinal artery resulted in persistent of hyperfluorescence in the late phase [Figure 2]. The optic disc fluorescence was unaltered. Fluorescein angiography was normal in the left eye. Optical coherence tomography indicated the presence of a serous retinal detachment impinging the macula [Figure 3]. Diagnostic workup that included coagulation studies, cardiovascular examination, electrocardiogram, echocardiogram, and Doppler evaluation of the neck vessels were within normal limits. Laboratory tests showed a hemoglobin level of 18.5 g/dL, an elevated hematocrit of 57.9%, red blood cell (RBC) count of 6,740,000/mm^3^, white blood cell (WBC) count of 10.790/mm^3^, platelet count of 217,000/mm^3^, and mean corpuscular volume was 78.5 fL. The arterial oxygen saturation level was 99%. A bone marrow biopsy showed hypercellularity with increased numbers of RBC precursors, WBC precursors, and platelets consistent with a diagnosis of polcythemia. Magnetic resonance imaging studies were normal. Moreover, the patient history was negative for migraine drug intake, and patient workup helped eliminate Horton’s disease and hypertension. On the basis of the diagnostic workup, laboratory evaluation, and patient history, the patient was diagnosed with polycythemia vera.

The patient was initially treated with therapeutic venesection followed by systemic hydroxyurea (500 mg four times daily tapered to 500 mg twice daily). However, visual acuity in the right eye did not improve despite treatment. After 6 months, visual acuity was 1/10 in the right eye.

## DISCUSSION

Polycythemia vera affects the hematopoietic stem cells and is characterized as a neoplastic marrow disorder^4^,^5^ and is associated with excessive proliferation of erythrocytes, leukocytes, and thrombocytes.^5^ Ocular complications vary and are often secondary to hyperviscosity and thrombosis.^4^ Despite decades of research, the etiology of polycythemia vera remains ambiguous.^6^

Ophthalmic effects of polycythemia vera related to hyperviscosity include transient ischemic attacks of the occipital cortex, transient monocular blindness,^2^–^4^ vaso-occlusive disease, and retinal hemorrhages.^7^ Inadequate cerebral perfusion may cause visual symptoms which can vary from hallucinations, amaurosis, scotoma, hemianopsia, and in the present case monocular blindness without associated pain.^4^,^7^

Isolated cilioretinal artery occlusions are rare and represents only 5% of all retinal artery occlusions.^2^–^4^ The precipitating factors for retinal artery occlusion are diverse, including embolism, carotid atherosclerosis, carotid arteriography,^2^ cardiac surgery,^2^,^5^ spasm during ergot overdose,^2^ and increased blood viscosity as present in polycythemia vera.^2^,^4^,^5^,^7^ The vulnerability of the cilioretinal artery is likely due to the specific anatomical bifurcation, which plays an important role in retinal blood flow,^5^,^7^ the lower perfusion pressure compared to the central retinal artery and lack of self-regulation of this perfusion pressure. In a healthy normal retina, the presence of a cilioretinal artery is clinically insignificant. However, in the presence retinal vascular occlusion, the effect of the cilioretinal artery can significantly affect visual morbidity.

To the best of our knowledge, isolated occlusion cilioretinal as an initial manifestation of polycythemia vera has not been previously reported in English peer review literature. Additionally, transient monocular visual blindness as an initial presentation of polycythemia vera has only been reported three times in the literature, indicating the rarity of this clinical presentation.^7^–^9^

Monocular visual loss due to polycythemia vera has been attributed arteriospasm, retinal artery occlusion due to arteriosclerosis, or platelet emboli.^2^ Considering that blood hyperviscosity likely plays an important role in this polycythemia meager number of reports of visual loss are surprising. The patient in this study experienced a sudden and severe decrease in visual acuity with characteristic retinal hallmarks of occlusion of the cilioretinal artery. The final visual prognosis after cilioretinal artery occlusion depends on the whether and to what extent this artery supplies the macula and in our patient the outcome was poor. Isolated cilioretinal artery occlusion as initial manifestation of polycythemia vera is rare and is an instance where emergent collaboration with the hematologist aids in the diagnosis and a potential reduction in visual morbidity.
